# Greater Cognitive-Motor Interference Among Patients After Anterior Cruciate Ligament Reconstruction Compared With Controls

**DOI:** 10.1177/03635465251322947

**Published:** 2025-03-04

**Authors:** Andrew Strong, Carl-Johan Boraxbekk, Jonas L. Markström

**Affiliations:** * Unit of Physiotherapy, Department of Community Medicine and Rehabilitation, Umeå University, Umeå, Sweden; † Department of Clinical Medicine, Faculty of Health and Medical Sciences, University of Copenhagen, Copenhagen, Denmark; ‡ Institute of Sports Medicine Copenhagen, Bispebjerg Hospital, University of Copenhagen, Copenhagen, Denmark; § Department of Neurology, Bispebjerg Hospital, University of Copenhagen, Copenhagen, Denmark; Investigation performed at Ume Ô University, Ume Ô, Sweden

**Keywords:** knee, ligaments, dual task, physical therapy/rehabilitation, return to sports, jump testing, cognition

## Abstract

**Background:**

Chaotic sporting environments require the performance of concurrent cognitive and motor tasks. A reduced capacity for either or both of the tasks when performed concurrently is known as cognitive-motor interference (CMi) and is believed to increase the injury risk. A greater susceptibility to CMi after a rupture of the anterior cruciate ligament (ACL) has been suggested to be caused by central nervous system adaptations, thus possibly contributing to high secondary ACL injury rates.

**Purpose:**

To investigate whether patients after ACL reconstruction (ACLR) demonstrate greater CMi than noninjured controls when adding secondary cognitive tasks to the drop vertical jump (DVJ) and explore the potential influence of sex on CMi.

**Study Design:**

Controlled laboratory study.

**Methods:**

A total of 40 (50% male) sports-active patients who had undergone ACLR (mean, 24.9 ± 16.1 months after surgery) and 40 (50% male) sports-active noninjured controls performed DVJs with and without secondary cognitive tasks targeting short-term memory, attention, fast decision-making, and inhibitory control. Outcomes included a letter position recall task and 3 motor variables: (1) correct action (landing or landing with a subsequent vertical jump), (2) relative jump height (relative between DVJs), and (3) relative peak vertical ground-reaction force (relative between DVJs). Participants also completed isolated cognitive tests (CANTAB) included as covariates in multivariate analysis.

**Results:**

Multivariate analysis of variance revealed that the ACLR group had greater CMi than the control group (*P* < .001), as manifested by more incorrect answers for the cognitive letter recall task (mean difference [MD], –13.3% [95% CI, –20.8% to –5.9%]; *P* < .001), more incorrect motor actions (MD, –7.5% [95% CI, −12.4% to –2.6%]; *P* = .003), and a reduced relative jump height (MD, –4.5% [95% CI, –7.9% to –1.2%]; *P* = .010). No difference in relative peak vertical ground-reaction force was found (MD, 2.8% [95% CI, –7.7% to 13.3%]; *P* = .59). Isolated cognitive outcomes did not affect these results, and there were no significant differences between male and female participants.

**Conclusion:**

Patients after ACLR showed greater CMi than noninjured controls, which was unrelated to isolated cognitive outcomes, thus indicating aberrant neurocognitive function.

**Clinical Relevance:**

Clinicians should consider cognitive and dual-task training and screening during ACL rehabilitation to better prepare patients for chaotic and uncontrolled sporting environments in which dual tasking is prevalent. Such interventions may help to reduce the risk of secondary ACL injuries.

The cognitive demands of sports are believed to impair concurrent movement control and thus increase the risk of injuries.^20^ Research concordantly has shown that performing cognitive and motor tasks simultaneously can lead to deficits in the outcomes of one or both tasks,^28,45^ a phenomenon known as cognitive-motor interference (CMi).^24^ Evidence also indicates that CMi is further increased after musculoskeletal trauma, such as an anterior cruciate ligament (ACL) injury.^30^ Return-to-sports screening after an ACL injury currently examines motor tasks in isolation, thus neglecting CMi. To improve the ecological validity of return-to-sports screening by closer resembling the demands of chaotic sporting environments, it has been suggested that neurocognitive and functional motor tasks should be combined.^5,16^

A rupture of the ACL results in the loss of sensory receptors found in the intact ligament,^1^ which may lead to reduced brain connectivity^9^ and to central nervous system adaptations.^29,31^ This theory is supported by some studies that have revealed atypical brain responses among patients with ACL injuries when performing isolated lower limb movements,^7,17,22^ although not for a lower limb proprioception task.^38^ Evidence of increased cognitive and cross-modal neural activity^6^ has nevertheless been theorized to reflect a heightened attentional focus on movements that may have been performed more automatically before an injury, leading to a greater susceptibility to CMi.^16^ A greater susceptibility to CMi is subsequently likely to result in poorer movement control during sports and may contribute to high secondary ACL injury rates.^32,43^

Greater CMi among patients with an ACL injury compared with controls was reported in a systematic review for postural control and gait.^30^ However, there is a lack of evidence comparing CMi between these groups for more athletic tasks such as hops or jumps. However, 2 recent studies did compare the biomechanical outcomes for unplanned landing between active soccer players with and without a previous ACL injury. One study found CMi for both groups, with movement patterns believed to increase the ACL injury risk, but no differences between groups.^2^ The other study found greater changes in landing mechanics such as greater vertical ground-reaction force (vGRF) and smaller knee flexion angles for those with ACL injuries compared with those without.^13^ Another recent study found that single-leg hop distance was similarly decreased for athletes with and without a previous ACL injury when a working memory task was performed concurrently.^15^ However, coordination variability was unchanged in only the leg with the ACL injury, suggesting cognitive control of the motor action after an injury.^15^ This limited and slightly contrasting evidence thus motivates further investigation in this area because greater CMi among patients with an ACL injury would justify adding simultaneous neurocognitive elements to return-to-sports screening.

Potential differences in CMi between sexes should also be considered, as female soccer and basketball players have an approximately 3 times higher risk of ACL injuries,^33^ show a more significant increase in the ACL injury rate,^4,35^ and may show differences in cognitive function.^21,46^ In support of this, a recent study by Lucia et al^25^ found that a 5-week cognitive-motor dual-task training intervention among basketball players resulted in different motor and cognitive improvements for male and female athletes as well as sex-dependent changes in preparatory brain activity for the cognitive task.

With this study, our objective was to examine the hypothesis that patients with an ACL injury display greater CMi than noninjured controls when secondary cognitive tasks are added to a drop vertical jump (DVJ). We also explored the potential influence of sex on CMi.

## Methods

This cross-sectional study, approved by the National Ethical Review Authority (2023-00342-01), was performed in a research laboratory. All participants gave prior written informed consent to partake in the study, and the study adhered to the ethical principles of the Declaration of Helsinki.

### Participants

This study involved 40 patients who had undergone ACL reconstruction (ACLR; 50% male) and 40 noninjured controls (50% male) (Table 1). Participants were recruited between April 2023 and April 2024 using a register from the orthopaedic clinic of a regional hospital in northern Sweden and via advertisements, local contacts, and word of mouth. Inclusion criteria were as follows: 15 to 36 years of age, a Tegner activity scale^41^ score ≥6 (of 10), returned to sports consisting of unpredictable and rapid directional changes performed on a weekly basis (eg, soccer, floorball, handball, volleyball, racket sports), confident of performing maximal hop and strength tests, a unilateral ACL injury, an ipsilateral hamstring tendon graft (standard national practice), maximum 5 years after ACLR, no concomitant injuries (ie, complete tear of any other knee ligament, major meniscal or articular damage), no severe ankle sprain within 6 months, and no other abnormality that would affect their ability to hop. The same relevant criteria applied to the controls, who were recruited from advertisements, local contacts, and word of mouth. The age range was selected based on 15 years being a common age that athletes in the relevant sports may begin competing at a higher level alongside adults. Our focus on sports characterized by unpredictable and rapid directional changes was based on ACL injuries mainly occurring in such sports.

**Table 1:** Participant Characteristics^*a*^

	ACLR	Control
Sex, male/female, n	20/20	20/20
Age, y	25.7 ± 5.5	22.0 ± 4.9
Height, m	1.72 ± 0.10	1.75 ± 0.09
Weight, kg	72.6 ± 11.5	72.3 ± 12.4
Body mass index, kg/m^2^	24.5 ± 2.7	23.5 ± 3.3
Time from ACLR to testing, mo	24.9 ± 16.1	—
IKDC score, %	82.9 ± 9.8	97.6 ± 4.3

a Data are presented as mean ± SD unless otherwise specified. ACLR, anterior cruciate ligament reconstruction; IKDC, International Knee Documentation Committee.

### Study Procedures

Participants completed the 2000 International Knee Documentation Committee (IKDC) subjective knee form and then the Cambridge Neuropsychological Test Automated Battery (CANTAB)^11^ on an iPad (Apple). Next, wearing sports clothing (their own shoes, short tights, and sports bra if female), they undertook a standardized warm-up (2 rounds of 6 body-weighted squats, 3 lunges per leg, and 3 squat jumps followed by 3-4 DVJ practice trials) and performed the DVJ without and with secondary cognitive tasks as described below. The same test leader (J.L.M.) instructed all participants.

#### Cognitive Tests (CANTAB)

Participants completed the following CANTAB tests in this order: Motor Screening (used as an introductory test), Reaction Time, Multitasking, and Paired Associates Learning (Appendix 1, available in the online version of this article). Overall, 3 key outcomes, one from each of the 3 latter tests, were used as covariates to control for cognitive performance (see the “Outcome Variables” section).

#### Jump Testing

**Figure 1.**
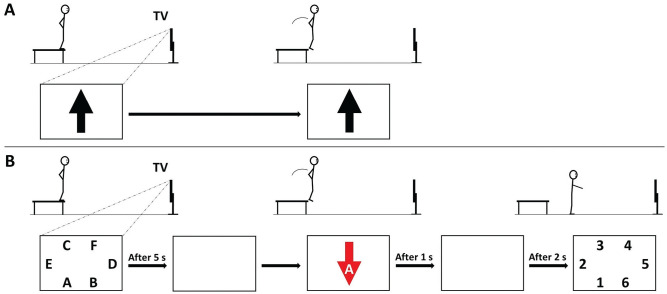
The drop vertical jump (DVJ) paradigm. (A) DVJ: While standing on the box, participants saw a black arrow pointing up or down on the screen (~5 m away) and then dropped down at a time of their choosing and performed the correct motor action. A black arrow pointing up indicated landing and immediately jumping as high as possible, while a black arrow pointing down indicated landing only. There were 3 trials conducted for each arrow in a randomized order, with a counterbalanced total such that upward- and downward-pointing black arrows were displayed 3 times each (n = 6). (B) DVJcog: Additional cognitive elements were added to challenge short-term memory, attention, fast decision-making, and inhibitory control. Participants first had 5 seconds to memorize the position of the letters A-F. Then, they dropped down from the box, and as soon as their heel lifted from the box, one of the arrows was shown with a letter inside for 1 second (element of fast decision-making). Alternatively to the black arrows, an upward- or downward-pointing red arrow indicated performing the opposite action compared with the black arrows (element of inhibitory control). They were to then memorize the letter, perform the correct motor action, and recall where the letter in the arrow was positioned (elements of attention and short-term memory). The order of the letters A to F and the letter inside the arrow were randomized between trials. There were 3 trials conducted for each arrow in a randomized order, with a counterbalanced total such that upward- and downward-pointing black and red arrows were displayed 3 times each (n = 12). Trials with an incorrect motor action were (unbeknownst to the participant) repeated after the 12 trials to achieve 3 successful trials for each arrow.

Participants performed DVJs without (DVJ) and with (DVJcog) secondary cognitive tasks (Figure 1) in a randomized order. The DVJ included preplanned motor actions, and the DVJcog involved unplanned motor actions. For both, participants stood on a 35 cm–high wooden box and then dropped down and approximately 50 cm forward, while visual cues (arrows) on a screen indicated whether they should only land or land and jump as high as possible. The following instructions adhering to an external focus of attention were provided before the jump: “Land softly and as quietly as possible, and if jumping after landing, push the ground away as hard as you can to jump as high as possible.” Detailed information is found in the Figure 1 caption.

### Instruments

Landing occurred on 2 force plates (1200 Hz; Model 9260AA [Kistler]) positioned side by side, with one foot on each. The force data were low-pass filtered at 50 Hz and synchronized with an 8-camera motion capture system (240 Hz; Oqus 300 [Qualisys]) recording the movements of passive reflective markers affixed on participants’ pelvis.

The arrows and letters during the DVJs were activated by a custom-made system that detected events using a laser-ranging time-of-flight sensor (VL53L1X; STMicroelectronics) placed on the box directed toward participants’ heels and a microcontroller (Uno; Arduino). The hardware communicated these events through a serial interface to software on a computer. The time between when the arrows appeared on the screen and participants landed was calculated using an optical sensor that registered when the arrow appeared and the initial detection of vGRF (>20 N for either force plate). For DVJcog, the arrows appeared at a mean of 280 ± 64 milliseconds before landing (variation due to drop strategy). The microcontroller firmware was written in proprietary language, and the computer software was written in C# language.

### Outcome Variables

The main outcomes are presented in Table 2. The test leader noted the correct number of letter position recall answers and correct number of motor actions during testing. The letter position recall answer was judged as correct if the position was correct, and the motor action was judged as correct if the action was correct and performed smoothly as one motion without pause. Jump height was calculated as displacement of the center of mass of the pelvis from standing to peak height during the DVJ using Visual3D software (Version 5.02.30; C-Motion). The peak vGRF was extracted during the first landing using Visual3D. The CANTAB outcomes were determined using associated software.

**Table 2:** Outcome Variables^*a*^

Outcome	Description
Jump testing	
Correct cognitive action	Percentage of correct letter position recall answers
Correct motor action	Percentage of correct subsequent motor actions during jump testing (ie, only land or land followed by maximal vertical jump in fluent movement)
Relative jump height	Mean difference in jump height for DVJ (black arrow pointing up) relative to DVJcog (red arrow pointing down); value <100% indicates lower jump height in DVJcog than DVJ
Relative peak vGRF	Mean difference in peak vGRF for DVJ (black arrow pointing up) relative to DVJcog (red arrow pointing down); value >100% indicates stiffer landing in DVJcog than DVJ and thus poorer adherence to instructions for soft landing
CANTAB	
5-choice reaction time (Reaction Time test evaluating processing and psychomotor speed)	Median duration for participant to release response button after being presented with target stimulus; calculated across correct assessed trials in which stimulus could appear in any 1 of 5 locations (in ms)
Multitasking cost (Multitasking test evaluating executive function and decision-making)	Difference between median latency of response (from stimulus appearance to button press) during assessed blocks in which 2 rules are used versus assessed blocks in which only a single rule is used; calculated by subtracting median latency of response during single task block(s) from median latency of response during multitasking block(s) (in ms)
Total errors (Paired Associates Learning test evaluating visual episodic memory)	Number of times that participant chose incorrect box for stimulus on assessment problems

a CANTAB, Cambridge Neuropsychological Test Automated Battery; DVJ, drop vertical jump without secondary cognitive tasks; DVJcog, drop vertical jump with secondary cognitive tasks; vGRF, vertical ground-reaction force.

### Statistical Analysis

We performed a power analysis based on data from a pilot study that we conducted in 2022 including the same DVJ dual-task paradigm used in this study, including 6 athletes with ACLR and 6 noninjured athletes. Analysis revealed an effect size (ES) of 1.12 for the cognitive task (ie, the letter position recall task) and indicated that 15 participants per group was required to achieve 90% power (independent *t* test, 1-sided hypothesis, 5% significance level, using mean and SD). Because the overall project included biomechanical analyses (not included in this study), we recruited 40 patients who had undergone ACLR (ACLR group) and 40 noninjured controls (control group) to also achieve power for biomechanical outcomes (Table 1). The 12 participants in the pilot study were not included in this study.

To examine the main objective, multivariate analysis of variance (MANOVA), including the 4 jump testing outcomes (Table 2), was performed to analyze CMi between groups and sexes (both included as factors). The same analysis, including the 3 CANTAB covariates in Table 2 (ie, multivariate analysis of covariance [MANCOVA]), was also performed to control for task-relevant cognitive ability. Multivariate normality and multicollinearity were evaluated by calculating the Mahalanobis distance and correlating the outcomes and covariates using the Pearson *r* coefficient, respectively. The Pillai trace was used,^40^ and the partial eta squared value as the ES was presented for main effects (0.01 = small; 0.10 = medium; 0.25 = large).^42^ Significant MANOVA and MANCOVA findings were further investigated using incorporated *t* tests for each outcome. The 3 CANTAB covariates were compared between groups using independent *t* tests after inspecting assumptions of normality and equal variance, also relying on the central limit theorem. The Statistical Package for Social Sciences (Version 23; IBM) was used for all analyses with a 5% significance level set a priori.

## Results

**Figure 2.**
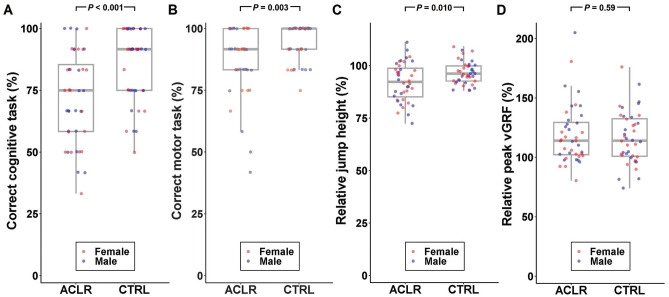
Individual data (dots) and boxplots for the 4 cognitive-motor interference (CMi) outcomes. (A) The percentage of correctly performed cognitive tasks (ie, letter position recall). (B) The percentage of correctly performed motor tasks (ie, only land or land followed by a maximal vertical jump). (C) Relative jump height (ie, the mean difference in jump height for DVJcog relative to DVJ in which a value <100% indicates a lower jump height for DVJcog). (D) Relative peak vertical ground-reaction force (vGRF; ie, the mean difference in peak vGRF for DVJcog relative to DVJ in which a value <100% indicates higher peak vGRF for DVJcog).

**Figure 3.**
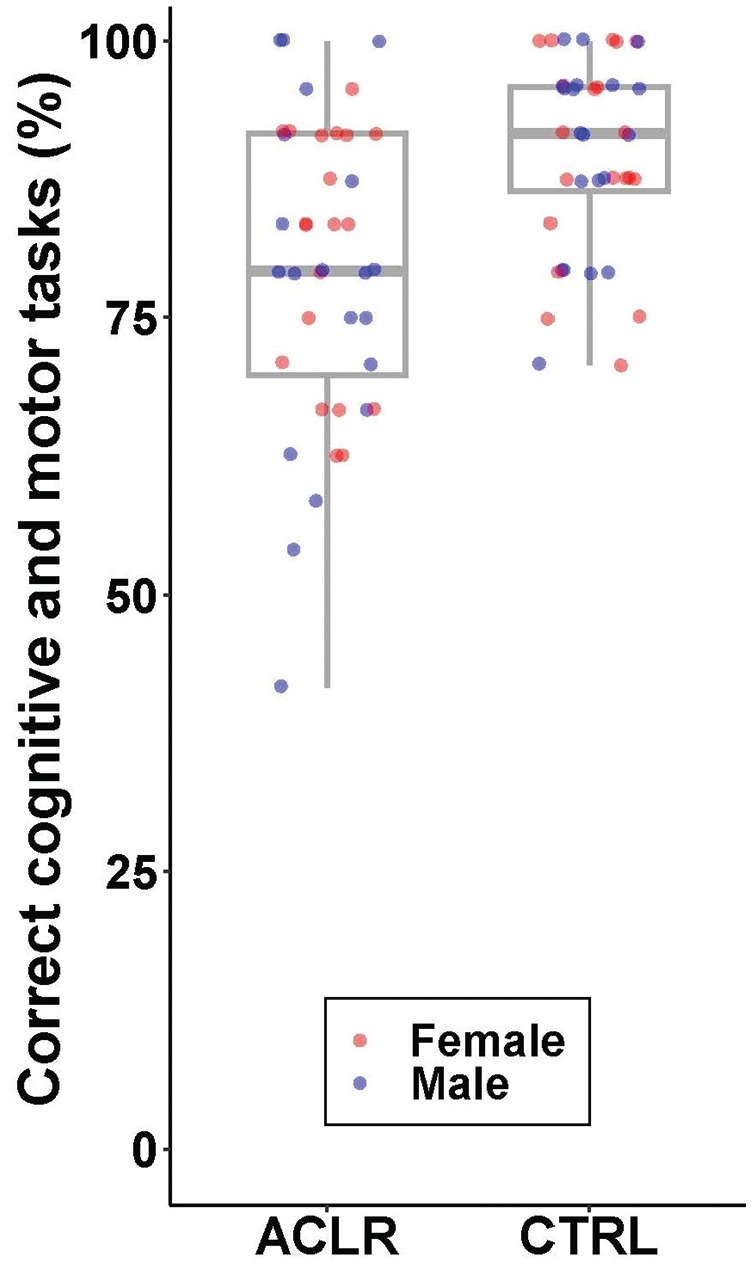
Combined results for the cognitive task (ie, letter position recall) and the motor task (ie, only land or land followed by a maximal vertical jump).

MANOVA revealed a significant main effect between groups (*F* = 5.53; *P* < .001; ES = 0.23 [medium]; power = 0.97) where the ACLR group had fewer correct trials for the cognitive and motor tasks (*P* < .001 and *P* = .003, respectively) and a larger relative decrease in jump height (*P* = .010) (Figure 2). No between-group differences were found for the relative peak vGRF (*P* = .59). Sex was not a significant factor (*P* = .67; ES = 0.03 [small]), and no interaction between groups and sexes was observed (*P* = .50; ES = 0.04 [small]). Figure 3 shows the percentage of correct trials for the combined cognitive and motor tasks, revealing that the lower values for the ACLR group were not attributed to a subset of participants but instead reflected fewer correct cognitive and motor actions of the group as a whole. There was no between-group difference in jump height during the DVJ (*P* = .24). CMi results were not associated with time after ACLR, which was a mean of 24.9 ± 16.1 months (Appendix 2, available online).

Regarding the CANTAB covariates, the ACLR group had a longer 5-choice reaction time (*P* = .001), but no differences were found for multitasking cost (*P* = .28) or total errors (*P* = .67) (Table 3). The between-group differences in CMi did not change when considering these covariates for MANCOVA. The main effect between groups was still significant (*F* = 3.95; *P* = .006; ES = 0.18 [medium]; power = 0.89), as were the cognitive outcomes (*P* = .001), motor outcomes (*P* = .027), and decreased relative jump height (*P* = .047).

**Table 3:** Results for Outcome Variables^*a*^

	ACLR	Control	MD (95% CI)
Correct cognitive action, %	71.9 ± 18.6	85.2 ± 14.7	−13.3 (–5.9 to −20.8)^*b*^
Correct motor action, %	86.9 ± 14.0	94.4 ± 7.2	−7.5 (–2.6 to −12.4)^*b*^
Jump height during DVJ, m	0.40 ± 0.09	0.42 ± 0.09	0.02 (–0.07 to 0.02)
Relative jump height, %	92.2 ± 9.2	96.7 ± 5.4	−4.5 (–1.2 to −7.9)^*b*^
Relative peak vGRF, %	119.3 ± 25.1	116.5 ± 22.1	2.8 (–7.7 to 13.3)
CANTAB (outcomes used as covariates)			
5-choice reaction time (Reaction Time test), ms	376 ± 33	352 ± 29	24 (10 to 38)^*b*^
Multitasking cost (Multitasking test), ms	181 ± 96	160 ± 74	21 (–17 to 59)
Total errors (Paired Associates Learning test), n	4.2 ± 4.3	4.6 ± 4.6	−0.4 (–2.4 to 1.6)

a Data are presented as mean ± SD unless otherwise specified. ACLR, anterior cruciate ligament reconstruction; CANTAB, Cambridge Neuropsychological Test Automated Battery; DVJ, drop vertical jump; MD, mean difference; vGRF, vertical ground-reaction force.

b Significantly different between groups at the .05 alpha level.

## Discussion

### Main Findings

Patients who returned to sports after rehabilitation from ACLR showed greater CMi than noninjured controls during DVJs when adding secondary cognitive tasks. This CMi was manifested in a greater number of incorrect actions on cognitive and motor tasks and a decreased relative jump height. Furthermore, isolated cognitive performance did not affect these findings, no significant differences in CMi were shown between the sexes, and no between-group difference in jump height during the DVJ without secondary cognitive tasks was found.

### Altered Neurocognitive Function After ACL Injury

Research evaluating neuromotor control among patients with an ACL injury generally supports the current opinion that an ACL rupture is a sensorimotor injury.^6,34^ Although brain imaging studies have provided valuable insights into neuroplasticity,^7,17,22^ such experiments are limited to simple motor tasks requiring minimal head movements to reduce data artifacts. These studies have also not considered CMi.

The existing literature investigating CMi after ACL injuries has either included only low-impact tasks^10,27,37^ or been limited by small sample sizes and mainly assessed alterations in landing mechanics.^2,13,14,18^ Our study included a high-impact task and a relatively large sample size equally divided between sexes with multiple cognitive and motor outcomes. The current study thus fills the knowledge gap by providing direct evidence of CMi after ACLR during a complex high-impact motor task.

Our result of greater CMi in the ACLR group is consistent with the previously mentioned systematic review for postural control and gait.^30^ Combined, these findings support current opinions that cognitive and motor tasks should be assessed in dual-task paradigms during ACL rehabilitation to better mimic sporting environments.^5,16^ Importantly, the poorer performance of our ACLR group was not explained by isolated outcomes of cognitive function (CANTAB). This further strengthens theories of altered neurocognitive function after an ACL injury^6,34^ and a greater susceptibility to CMi,^16^ which seems to affect both sexes equally. Although we did not observe any differences in CMi between the sexes, further research comparing male and female patients in this area is warranted because of the higher ACL injury risk for female patients^3^ as well as differences in cognitive function^21,46^ and cognitive-motor dual-task training outcomes.^25^

The central capacity sharing model states that limitations in information processing capacity during dual tasks require the redistribution of resources between the 2 tasks.^12^ The participants in our ACLR group generally prioritized the motor task over the cognitive task, as evidenced by the greater number of correct trials, but still demonstrated a lower relative jump height than the controls (Table 3). One explanation could be that they used more cognitive resources than the controls for landing because of their injury. Prioritizing motor actions at the expense of cognitive performance among patients with an ACL injury is also supported by previous research.^10,30^ Another explanation concerning both groups could be the competitive element. We noted that participants were often aware when they performed incorrect motor actions by showing emotion, often by cursing or sighing. For the cognitive letter recall task, however, they were usually unsure whether they answered correctly (confirmed when asked after completing the test session) and were thus not equally emotive. The influence of outcome feedback during dual tasking therefore warrants consideration in future studies.

### Isolated Cognitive Performance

Our ACLR group revealed similar results to the control group for multitasking cost (Multitasking test evaluating executive function and decision-making) and total errors (Paired Associates Learning test evaluating visual episodic memory) (Table 3). The total errors were also normal for both groups compared with the CANTAB normative data for adults in which the ACLR group was better than 71.2% (mean standard score: 0.82) and the control group was better than 68.1% (mean standard score: 0.72). These cognitive outcomes thus did not explain the greater CMi for our ACLR group. This is supported by recent findings that motor-cognitive function is unrelated to independently assessed motor and cognitive skills among noninjured sports students.^44^

The 5-choice reaction time of our ACLR group was, however, 7% (95% CI, 3%-11%) longer than that of our control group. While this outcome did not affect the CMi results, a longer reaction time and processing speed during sports could increase the injury risk.^39^ Greater motor planning and response inhibition during a reaction time task involving seated kicking actions among patients after ACLR have been reported previously, although more errors in response selection rather than reaction time was observed compared with controls.^36^

Additionally, neurocognitive impairments have been observed among male athletes with ACLR who both failed and passed return-to-sports criteria.^23^ Although the implications of such deficits on the injury risk are unclear, poorer visual-spatial memory among male athletes has been found to be associated with an increase in peak knee valgus angles when performing a sidestep cut while dribbling a soccer ball compared with dribbling without a soccer ball.^26^ Given that a greater knee valgus angle is considered an ACL injury risk factor,^20^ neurocognitive impairments seem an important consideration for the injury risk.

### Clinical Implications

Our findings support theories of neurocognitive deficits increasing the susceptibility to CMi among patients after ACLR. These results may help to explain the high risk for secondary ACL injuries, despite extensive rehabilitation and return-to-sports testing,^32^ given that ACL injuries mainly occur in noncontact situations when the athlete’s attention is occupied.^20^ To improve current rehabilitation practices, integrating cognitive aspects or dual tasks would better resemble the unpredictable nature of sporting environments and may help to reduce the negative effects of CMi, including a higher risk for secondary injuries.^16,19^ This could involve adding visual stimuli to a high-impact task, such as hopping or running, in which the athlete’s perception of the visual stimuli is required to guide the motor action, while they concurrently perform a working memory task. The reviews of Chaaban et al^5^ and Grooms et al^16^ provide further examples. Such training should be individually adapted and evaluated while considering the substantial between-person variation in cognitive and motor performance (Figure 2). To improve assessments of readiness for return to sports after an ACL injury, our findings support the suggestion that functional screening tests should include secondary cognitive demands in which both cognitive and motor performance are evaluated.^5,16^ Whether such training and screening improve CMi and result in a safer return to sports for patients with a history of ACLR is, however, an understudied area that warrants further investigation.

### Strengths and Limitations

The generalizability of our results is influenced by 4 factors. First, our participants were involved in different sports, although all consisted of rapid directional changes and thus targeted the overall population of interest. Second, ACL rehabilitation was not standardized, which limits our understanding of its influence on the outcomes but enhances the generalizability. Third, the time between ACLR and testing varied; however, time since ACLR did not correlate to any of the outcomes (Appendix 2). Fourth, all patients in the ACLR group had a hamstring tendon graft (national practice), which restricts the extrapolation of our results to patients with a history of ACLR using other grafts.

We lack preinjury data to infer causality that an ACL injury results in greater CMi, and thus, we cannot be sure whether our participants showed heightened CMi also before their ACL injury. Longitudinal studies assessing CMi before and after an ACL injury are therefore encouraged. We also did not screen our participants for learning disabilities, neurodivergent conditions, or medications. Our attempt to control for isolated cognitive performance using the CANTAB is nevertheless a methodological strength of our study. The ceiling effect observed for some participants for the combined cognitive and motor task results (Figure 3) does, however, limit our understanding of the “true” between-group difference, which is likely greater than that observed here. Letters from a familiar alphabet may have facilitated different memory techniques via, for example, verbal mediation to create fewer blocks of information to memorize.^8^ Using symbols or figures without meaning to the participant could increase the difficulty level and avoid a ceiling effect, thus enhancing validity. The motor task can also be made more difficult by adding additional options, such as directional jumps. Further, additional trials would decrease the probability of perfect scores for the cognitive and motor tasks but may induce fatigue, which could affect some outcomes. Additional trials would nevertheless only increase our observed between-group differences, given that 3 patients (7.5%) in the ACLR group and 8 controls (20.0%) achieved 100% correct performance for the dichotomous (correct/incorrect) cognitive and motor tasks.

## Conclusion

Patients with a history of ACLR showed greater CMi than noninjured controls when secondary cognitive tasks were added to a DVJ, as revealed by poorer performance for both integrated cognitive and motor tasks. Isolated cognitive outcomes assessing reaction time, multitasking cost, and visual episodic memory errors did not affect these results, and no differences in CMi were shown between male and female participants. Our findings strengthen the viewpoint that an ACL injury is a sensorimotor injury.

## Supplemental Material

sj-pdf-1-ajs-10.1177_03635465251322947 – Supplemental material for Greater Cognitive-Motor Interference Among Patients After Anterior Cruciate Ligament Reconstruction Compared With Controls Supplemental material, sj-pdf-1-ajs-10.1177_03635465251322947 for Greater Cognitive-Motor Interference Among Patients After Anterior Cruciate Ligament Reconstruction Compared With Controls by Andrew Strong, Carl-Johan Boraxbekk and Jonas L. Markström in The American Journal of Sports Medicine
