# Supplementary material for: Greater Cognitive-Motor Interference Among Patients After Anterior Cruciate Ligament Reconstruction Compared With Controls
Source: Am J Sports Med. 2025 Mar 4;53(5):1041–9. doi: 10.1177/03635465251322947 (PMC11951357; doi:10.1177/03635465251322947)

Greater Cognitive-Motor Interference Among Individuals After Rehabilitation from Anterior Cruciate Ligament Reconstruction Compared with Controls

Appendix 1: Description of the CANTAB tests

The following CANTAB tests were performed in the presented order:

Motor screening task – evaluates sensorimotor function and comprehension

https://www.cambridgecognition.com/cantab/cognitive-tests/attention/motor-screening-task-mot/

Description: Coloured crosses are presented in different locations on the screen, one at a time. The participant must select the cross on the screen as quickly and accurately as possible. This is recommended as an introductory test and we used it as such.

Reaction time – evaluates processing and psychomotor speed

https://www.cambridgecognition.com/cantab/cognitive-tests/attention/reaction-time-rti/

Description: The participant must press and hold a button at the bottom of the screen. Circles are presented above (one for the simple mode, and five for the five-choice mode.) In each case, a yellow dot will appear in one of the circles, and the participant must react as soon as possible, releasing the button at the bottom of the screen, and pressing inside the circle in which the dot appeared.

Multitasking test – evaluates executive function and decision making

https://www.cambridgecognition.com/cantab/cognitive-tests/executive-function/multitasking-test-mtt/

Description: The test displays an arrow which can appear on either side of the screen (right or left) and can point in either direction (right or left). Each trial displays a cue at the top of the screen that indicates to the participant whether they have to select the right or left button according to the "side on which the arrow appeared" or the "direction in which the arrow was pointing". In some sections of the task this rule is consistent across trials (single task) while in others it may change from trial to trial in a randomised order (multitasking). Using both rules in a flexible manner places a higher demand on cognition than using a single rule. Some trials display congruent stimuli (e.g. arrow on the right side pointing to the right) whereas other trials display incongruent stimuli, which require a higher cognitive demand (e.g. arrow on the right side of the screen pointing to the left).

Paired associates learning – evaluates visual episodic memory

https://www.cambridgecognition.com/cantab/cognitive-tests/memory/paired-associates-learning-pal/

Description: Boxes are displayed on the screen and are "opened" in a randomised order. One or more of them will contain a pattern. The patterns are then displayed one at a time in the middle of the screen and the participant must select the box in which the pattern was originally located. If the participant makes an error, the boxes are opened in sequence again to remind the participant of the locations of the patterns. The number of boxes gradually increases until 8 boxes are shown for the participants with patterns to remember.

Appendix 2: Scatterplots for the CMi outcomes and time post-ACLR

The scatterplots show no association between time post-ACLR and number of correct cognitive tasks (top left), number of correct motor tasks (top right), relative jump height (bottom left), or relative peak vertical GRF (bottom right). This means that the cognitive-motor interference (CMi) outcomes are not affected by time post-ACLR within the time frames included in our sample of 40 individuals with ACLR.

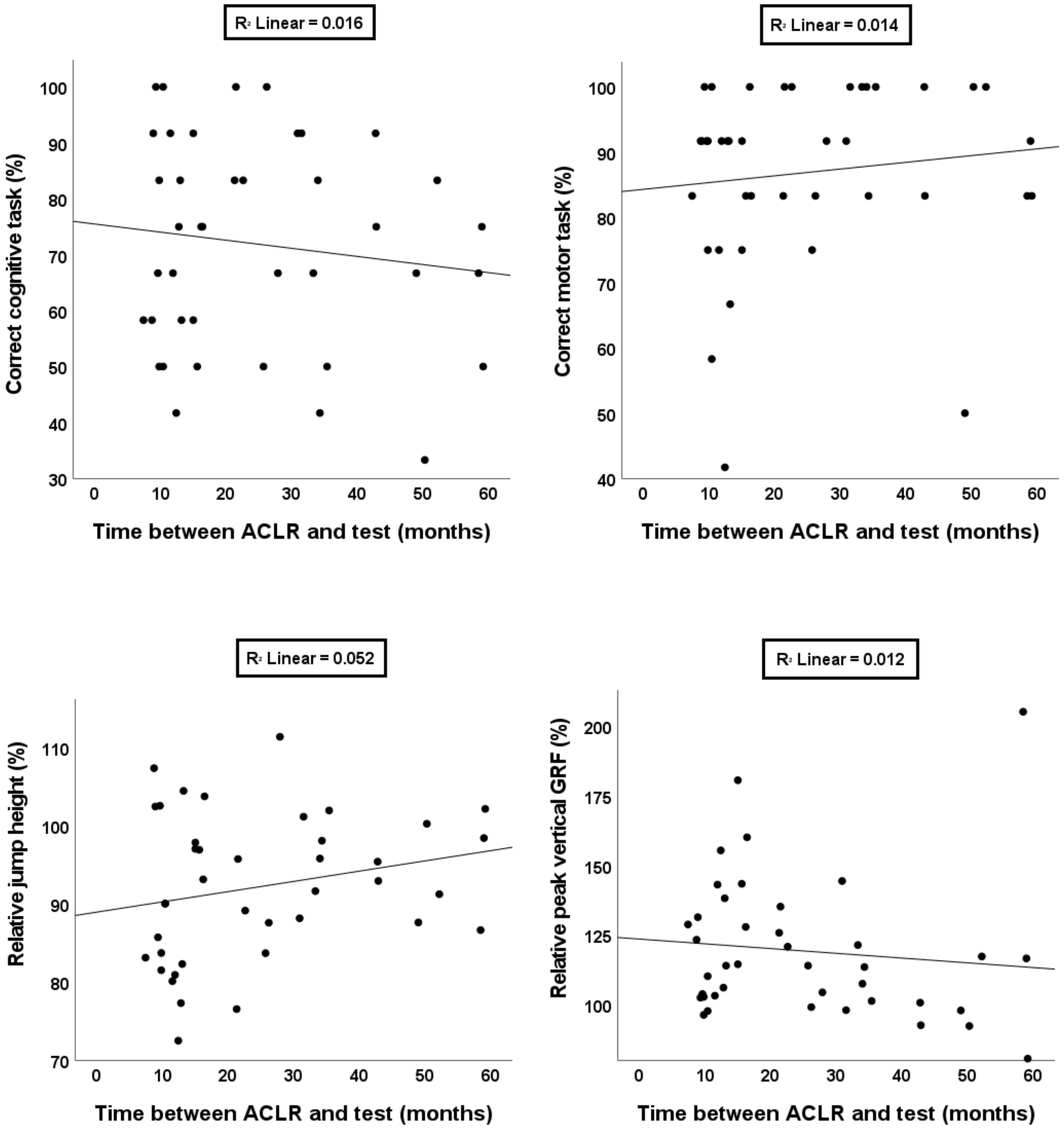

Supplement: sj-pdf-1-ajs-10.1177_03635465251322947 – Supplemental material for Greater Cognitive-Motor Interference Among Patients After Anterior Cruciate Ligament Reconstruction Compared With Controls [file sj-pdf-1-ajs-10.1177_03635465251322947.pdf]
